# Consumer-Related Antecedents of Waste Behavior in Online Food Ordering: A Study among Young Adults in China

**DOI:** 10.3390/foods11193098

**Published:** 2022-10-05

**Authors:** Li Jia, Yaoqi Zhang, Guanghua Qiao

**Affiliations:** 1 College of Economics and Management, Inner Mongolia Agricultural University, Hohhot 010000, China; 2 School of Forestry and Wildlife Sciences, Auburn University, Auburn, AL 36849, USA

**Keywords:** theory of planned behavior, food waste, consumption behavior, price consciousness, over-consumption

## Abstract

Food waste in the catering industry currently accounts for almost half of the total food waste in China and entails a large amount of land, water, and labor costs, in addition to the carbon footprint’s impacts on climate change. Under the background of increasing food consumption and waste from online catering, this study investigates the factors influencing the food waste behaviors (FW) of online food ordering in China and provides policy recommendations for food waste reduction. Using survey data from 482 consumers, we constructed a theoretical framework and examined the influence path of each factor using structural equation modeling (SEM) and a bootstrap test. The results showed that young consumers without farming experience and females wasted more on ordering food online. The more frequently the consumer ordered, the more they wasted. The level of consumers’ perceived behavioral control (PBC) was found to be lower than other factors, indicating that it was difficult for consumers to reduce food waste. Attitudes toward behavior (ATT), subjective norm (SN), PBC, and price consciousness (PC) were all positively related to behavioral intention to reduce food waste (BI). PBC and BI were negatively related to FW, and over-consumption behavior (OC) was positively related to FW. BI had a mediating effect on the paths of ATT, PBC, and PC to FW, but the pathway through which PC influenced FW was primarily through BI or PBC, not OC. In our research, BI had no mediating effect between SN and FW. Ultimately, our findings inform some policy recommendations to help nations, restaurants, food-ordering platforms, and consumers reduce waste.

## 1. Introduction

Although the world’s food production has rapidly increased over the past 50 years, food shortages remain a problem, with a total of 720 million to 811 million people around the globe facing hunger in 2020. This number marks an increase of about 118 million from 2019 [1]. In addition, the global agri-food system has been affected by multiple risks in recent years, including natural disasters, extreme climate change, and the ongoing COVID-19 pandemic [2]. It was noted that the international community may face difficulties achieving the Sustainable Development Goals by 2030 [3]. A total of 1.3 billion tons of food are lost and wasted globally each year [4,5]. Developing countries are likely to be the key players in determining the global food waste situation by the mid-21st century, and without major policy adjustments and behavioral changes, global per capita food waste will double by 2050 [6]. Food waste means large economic losses [7,8,9,10]. Accordingly, a reduction in waste has a positive impact on economic indicators such as import and export volumes and price indices of major agricultural products [11]. In addition, food waste creates significant resource and environmental costs. The ecological footprints [12], water footprints [13], and carbon footprints [14] of wasted food throughout its life cycle have increased environmental stress in regions where water and arable land are scarce, which means an inefficient use of valuable resources and negative pressure on global climate change.

Food loss and waste occur at different points in the food supply chain, from production, processing, storage, and distribution to food waste at the end of the food supply chain [4]. Here, we define food waste as the decline in food quantity at the end of the food supply chain (retail and consumption) caused by subjective consumer factors [15]. Food waste that occurs at the end of the supply chain has all the resource–environmental costs of growing, harvesting, processing, packaging, storing, transporting, and retailing food and can be generated at the household level or in food service industries such as the catering industry [16], which accounts for nearly 13% of total food waste [17]. The situation, however, is becoming worse. For China, food waste in the catering sector has become a concern [18,19], with studies reporting that urban catering in China wasted about 17–18 million tons of food per year in 2015, representing close to 3% of China’s food production. The amount of waste in large restaurants and campus canteens is staggering, and more than 30% of food in campus box lunches is discarded [20]. The Anti-Food Waste Law of China was enacted on April 29, 2021, to help society address the food waste situation in the catering industry and restrain stakeholder behavior through laws to reduce food waste. In the catering industry, online food ordering has increasingly prospered in recent years. Chinese online food ordering users totaled 456 million in 2020, with the business of online food ordering revenue reaching 811.94 billion Chinese Yuan. Moreover, individuals aged 20–35 years old are becoming the main consumers of online food ordering [21].

Food waste behavior is influenced by multiple factors such as personal characteristics [22,23], regional culture [24,25], and socio-economic conditions [26]. There remains a paucity of research on the fast-growing segment that orders food from restaurants for consumption through the use of delivery apps [27]. This gap needs to be addressed since online food ordering has become a prominent avenue of ordering food since the COVID-19 pandemic and is already considered to be a key food waste generator globally [28]. For online food consumption, the food supply system, including the platform, merchant sales strategy, food delivery method, food packaging form, food consumption area, etc., is more complex and diverse than the systems of other food consumption methods. Additionally, the information asymmetry in the food consumption process may introduce more food waste problems [29]. Based on the psychological factors of consumers and the specificity of the online food ordering context, we need to consider the wasteful behaviors caused by consumers’ personal factors and the excessive purchasing behaviors caused by commercial activities such as portion size, starting delivery amount, and full discount activities. We should also consider how to promote sustainable consumption in online food ordering among consumers.

This paper uses an extended theoretical framework to explore the causes of food waste behavior in online ordering among young consumers and to provide a reference for interventions and policy development for reducing food waste and promoting sustainable food consumption in the future. We focus on the psychological factors of consumers, including attitude, subjective norm, perceived behavioral control, and price consciousness. We also induced the over-consumption behaviors caused by commercial activities such as portion size, starting delivery amount, and full discount activities. The intermediate variables of behavioral intention to reduce food waste and over-consumption behavior provide important pathways between the independent and dependent variables. Our paper examines the influence path of each factor via Structural Equation Modeling (SEM) and a bootstrap test. We sought to determine how these independent variables influenced food waste behavior through these mediating variables. For the first time in the literature, our study provides a detailed analysis of how consumer psychological factors influence food waste behavior in online food ordering through the behavioral intentions to reduce food waste and over-consumption behavior. The results will inform suggestions for interventions to reduce food waste.

## 2. Literature Review and Hypothesis Development

### 2.1. Food Waste Behavior

A recent authoritative study showed that most environmental footprints associated with food loss and waste are produced at the consumption stage and that the carbon footprint associated with out-of-home waste is substantially greater than that of household food waste [17]. Food waste at the consumption end is caused by subjective consumer factors and is the result of a combination of multiple factors, such as the personal characteristics of consumers, regional culture, and economic circumstances. Food waste is further associated with different consumption behaviors and drivers in residential households, various types of restaurants, and public cafeterias. Exploring and scientifically analyzing the drivers of food waste behavior is essential to promote sustainable food consumption globally [30].

The occurrence of food waste may be related to the food’s opportunity cost, and consumers may not have a strong incentive to avoid food waste if food expenditures are only a small part of total household expenditures [31]. Food waste is income-elastic, with income growth significantly increasing the amount of food waste among residents [32]. Current research on factors influencing food waste mainly relate to household food waste behaviors. Existing studies suggest that, in addition to demographic factors [33], purchase planning (excessive or impulsive shopping) [34], understanding of labels [35], handling of food [36], household storage and cooking habits [37], and dietary knowledge [38] have significant effects on food waste behavior. Contextual factors are considered in studies related to food waste in the restaurant industry, such as a study on the food waste behaviors of dining out in Lhasa that showed how travel status significantly influenced waste behavior [39,40]. Plate size [16] and information interventions [41], among others, can also correlate to different waste levels.

In addition, consumer psychological factors are gradually gaining attention. For example, public environmental knowledge was introduced as an influencing factor in a study of the prerequisites for consumer involvement in reducing food waste in restaurants in Poland [42]. Since food waste is related to income and price, Price consciousness was also taken into consideration [43]. When food is consumed, consumers’ subjective norms [37], resource-environmental awareness [42], perceived behavioral control [44], and several other psychosocial factors [45] can influence food waste behavioral intentions and thus impact food waste behavior.

Many policy initiatives have been used around the world to reduce food waste, in addition to legislation and donations, with controversial effects [46,47]. Countries around the world have also introduced regional systems to reduce food waste, such as the food waste hierarchy suggested by the UK government (Available online: https://www.gov.uk/government/publications/food-and-drink-waste-hierarchy-deal-with-surplus-and-waste/food-and-drink-waste-hierarchy-deal-with-surplus-and-waste, accessed on 30 July 2022) and the Clean Your Plate campaign in China. In addition, there is now heavy use of lean techniques and digitalization to reduce food waste. Luca et al. proposed an approach to reduce out-of-home food waste in Italy by combining food surplus management and digital solutions with profitable business model innovation. The results supported the need for companies to invest in innovation and digital solutions to reduce food surplus and waste [48]. The implementation of an Internet of Things-based food waste tracking system can help identify food waste hotspots [49], and a categorization scheme for digital food waste technologies (forecasting, waste analysis, redistribution, and measures catalog) can also be used as a food-waste-prevention technology [50]. Lean management methods for food services can not only achieve efficient operations but also potentially eliminate food waste, based on three case studies from Poland [51]. Thus, such methods are worth promoting in the restaurant industry.

Since comprehensive municipal public policies and all consumer behaviors are important to reduce food waste [52], we require innovations based on technology and the expansion of existing technologies to develop strategic measures for changing the behaviors of producers, retailers, and consumers [53]. Clear regulations, policies, and strategies could be more effective in reducing food waste than financial measures [54]. The development of these measures and regulations requires an in-depth investigation into the motivations of consumers’ food waste and a targeted approach to influence consumer behaviors. Our paper focuses on the situation of consumer food waste in online food ordering in China and seeks to explore the current state and its drivers.

### 2.2. Theory and Hypotheses

**Figure 1:**
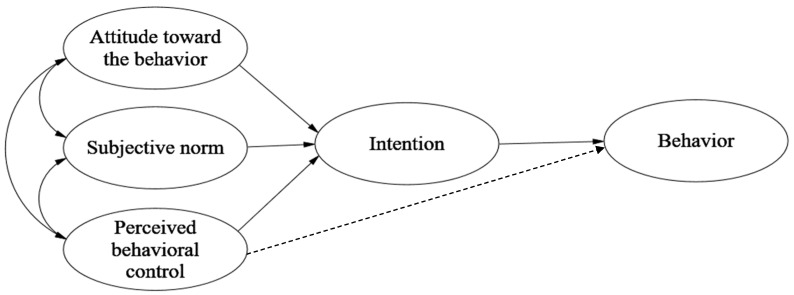
Theory of planned behavior. Source: Ajzen (1991, p. 182). [55].

Theory of planned behavior (TPB) was developed by Icek Ajzen (1991), which argues that individual behavioral intention (BI) is influenced by attitudes toward behaviors (ATT), subjective norms (SN), and perceived behavioral control (PBC). Ajzen believes that factors that can influence behavior indirectly influence behavior through behavioral intention [55] (The framework was shown in Figure 1). TPB has been widely used in various research fields related to personal behavior, such as tourism [56], environmental protection [57,58], education [59], business management [60], and consumer behavior [61,62]. Since food consumption behavior is influenced by one’s attitudes, social context, and sense of control, this measure has also been used to explain consumers’ food waste behavioral intentions [34,37,63]. Individual behavioral intentions are key determinants in predicting individual behavior, which is well supported by studies on household food waste behavior [34,64].

Attitudes are the positive or negative feelings that individuals hold about their behaviors [55]. Attitudes to reduce food waste mainly involve economic attitudes (buying less food to save money and reduce costs), environmental attitudes (being environmentally friendly and low carbon and reducing environmental pollution such as that from landfills), and moral attitudes (reducing world hunger and helping the needy) [65], together leading to the experience of negative emotions when food is wasted [66]. Although some studies have argued that consumers rarely associate food waste with the environment (e.g., pollution and carbon emissions) and tend to be more concerned about price [67], reducing food waste is also a pro-environmental behavior that promotes ecological awareness by increasing green consumption among consumers.

The subjective norm refers to the psychological tendency of consumers to be influenced by other factors such as social pressure [55]. Consumers’ subjective norms about food waste generally relate to perceived social pressures to perform, or not perform, food waste behaviors, stemming from the perceptions and practices of the government, school, family, and friends regarding food waste—especially from the perceptions and practices of those who have the greatest influence. Studies have shown that the subjective norm can significantly reduce food waste behavior in household [37,66] and dining-out situations [22], while some studies have shown the opposite results [63,68]. These differences may stem from specific and different populations and contexts, the difficulty of measuring food waste behavior, and consumers’ masked self-reporting. Consumers’ social networks may also have a significant impact on their food consumption and waste behavior [66]. For online food ordering, whether subjective norms have a significant impact on food waste behavior is less well known and worthy of further exploration. While Chinese traditional culture praises thrift and frugality and not wasting food, hospitality and generosity are also highly appreciated. While opposition to wasting food at the national, family, school, and society levels generates social pressure on food wasteful behaviors, ordering more food than desired and being reluctant to take leftovers home are very common behaviors while dining out and lead to food waste [18].

Perceived behavioral control refers to the difficulty of accomplishing a particular behavior and is mainly dependent on factors such as time, money, experience, and information. The perceived behavioral control of consumers’ food waste behavior relates to consumers’ perceptions of their ability to control their amount of food waste [64]. Further, perceived behavioral control significantly influences consumers’ behavioral intentions to reduce food waste [69] and food waste behavior [37]. For instance, unpredictable meal sizes and large packaging can lead to lower levels of perceived behavioral control [70], which, in turn, promotes food waste. In online food ordering consumption, consumers’ behavioral intentions to reduce food waste and their eventual waste behaviors are influenced by various factors such as time, the amount of the meal, and the conditions for disposing of leftovers.

Based on the analyses above, hypotheses H1–H5 are proposed:

**H1.**  *Attitude (ATT) has a significant positive effect on behavioral intention to reduce food waste (BI)*.

**H2.**  *Subjective norm (SN) has a significant positive effect on behavioral intention to reduce food waste (BI)*.

**H3.**  *Perceived behavioral control (PBC) has a significant positive effect on behavioral intention to reduce food waste (BI)*.

**H4.**  *Perceived behavioral control (PBC) has a significant negative effect on food waste behavior (FW)*.

**H5.**  *Behavioral intention to reduce food waste (BI) has a significant negative effect on food waste behavior (FW)*.

Based on a literature review and our interviews with consumers, price consciousness and over-consumption behaviors were included in our consideration. Young consumers are often prone to over-consumption behaviors, which may be related to special dietary preferences and/or merchandising aspects. Food purchase planning is an important part of food consumption, and good planning can significantly reduce food surplus. Over-consumption and impulse purchases (unplanned purchases) due to promotions and discounts are sources of food surplus and an important factor in food waste generation [45,68]. When consumers order food online, they are often unable to successfully purchase items below the minimum delivery amount, which is typically 15–20 (CNY), and will thus add more unwanted food to meet that minimum amount. In addition, many online merchants and network platforms will offer full discounts or coupons if consumers purchase more than a certain amount, inducing consumers’ over-consumption behaviors and a food surplus.

Over-consumption behavior towards food is also related to consumer price consciousness [34,43], which positively influences shopping plans. Consumers with strong price consciousness will follow their original plans very closely for food purchases [64,71] and are less likely to engage in over-consumption behaviors. Such consumers also tend to show stronger behavioral intentions to reduce food waste due to stronger price consciousness [72]. As such consumers perceive a lack of leftovers as valuable, they do not throw leftover food away but instead use that food, leading to greater perceived behavioral control to reduce waste.

Based on the analyses above, hypotheses H6–H8 are proposed:

**H6.**  *Price consciousness (PC) has a significant positive effect on behavioral intention to reduce food waste (BI)*.

**H7.**  *Price consciousness (PC) has a significant negative effect on over-consumption behavior (OC)*.

**H8.**  *Price consciousness (PC) has a significant positive effect on perceived behavioral control (PBC)*.

**H9.**  *Over-consumption behavior (OC) has a significant positive effect on food waste behavior (FW)*.

### 2.3. Model Structure

**Figure 2:**
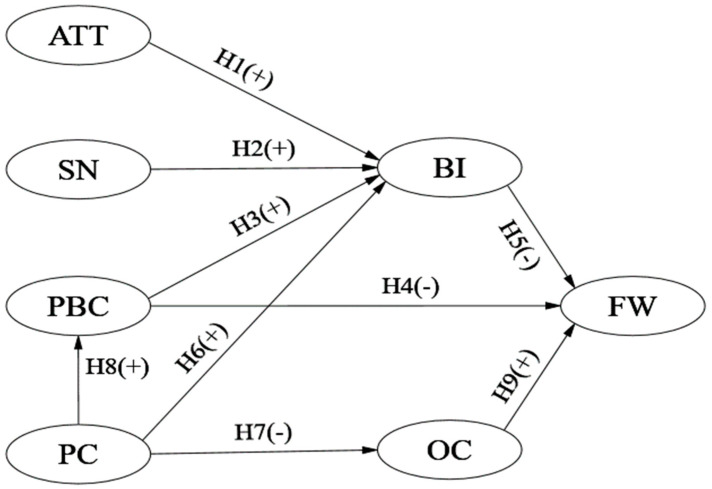
Theoretical hypothesis model.

Since TPB is a theory that is compatible with other predictors [55], such as personal norms [63,73], planning habits [67], environmental concerns [69], and injunctive norms [45], and can be adjusted by adding other factors to the model [74], some scholars have combined TPB and other variables and found that the predictive power of the integrated model was greatly improved. For example, one study analyzed food waste behavior in German restaurants [63]. In this paper, based on the theory of planned behavior, two variables, price consciousness and over-consumption behavior, are introduced to explore the motives underlying consumers’ food waste behavior. All variables in our study were closely related to behavioral intention and behavior to reduce food waste and were not related to general consumer explanations. For instance, consumers’ attitudes to reduce food waste (ATT) mainly involve economic attitudes (buying less food to save money and reduce costs), environmental attitudes (being environmentally friendly and low carbon and reducing environmental pollution such as that from landfills), and moral attitudes (reducing world hunger and helping the needy), together leading to negative emotions when such consumers waste food. These interpretations determined the design of our questionnaire to measure consumers’ attitudes towards food waste reduction. The comprehensive theoretical framework is shown in Figure 2.

## 3. Materials and Methods

### 3.1. Sample Selection

Studies on consumer food waste behaviors in China have mainly focused on first-tier cities [18,75] and tourist cities [39,40]. There are also studies related to food waste in university cafeterias across China [76]. However, very few studies have analyzed less-developed regions. A cluster analysis of the per capita consumption of major food items by urban residents in various regions of China (source: National Bureau of Statistics 2021) revealed that the per capita consumption of poultry, meat, eggs, milk, and aquatic products in less-developed regions (62.2 kg) was lower than that in developed regions (81.2 kg). To understand the food waste behaviors and motivations of young consumers in less developed regions, we selected college students from the Inner Mongolia Autonomous Region, China, as the research sample.

In 2021, 54 colleges and universities were located in Inner Mongolia Autonomous Region, with 29 in Hohhot and Baotou, whose students account for 67.6% of the total students in the region. In this study, 8 universities and 4 higher vocational colleges in Hohhot and Baotou were selected and studied in groups from September to December 2021, using the random sampling method. We distributed 528 questionnaires throughout the research period. After removing invalid respondents, we retained 482 valid respondents, with a rate of 91.29%. As this paper required structural equation analysis, the sample size was determined by a set amount of parameters according to the data requirements of SEM [77]. The details are shown in Table 1.

### 3.2. Questionnaire

The survey was conducted using a WeChat scan code in universities. A pre-survey was conducted in May 2021, and after a review and adjustment, the formal survey was conducted from September to November 2021. In collecting the data, we prompted respondents that food waste refers to avoidable waste (excluding bones, peels, seasonings, and soups).

The questionnaire was divided into three parts. The first part collected information on consumers’ estimations of the amount of food they wasted from online food ordering (measured using a seven-point Likert scale) along with some background information (cost and ordering frequency). The second part collected information on the measured items for each variable involved in the study model (using a seven-point Likert scale). The measurements of latent variable items and reference sources are shown in Table 2. The third section collected information on consumer demographics and social characteristics, including gender, grade, family sources, monthly household income, and whether they were vegetarians. We used SPSS 25.0 and AMOS 23.0 to estimate both the measurement model and the structural model. The data were tested for missing values, outliers, and normality before proceeding to further analysis.

## 4. Results

### 4.1. Descriptive Analysis

The demographic information of the sample is shown in Table 1. The meals that respondents ordered for breakfast, lunch, and dinner in our survey accounted for 11.2%, 56.2%, and 32.6% respectively, and the cost of online food ordering was mainly concentrated around 10–30 RMB. In the estimation of food wasted from the last meal ordered online, 49%, respondents reported that they wasted more than 20%, and 8.3% of respondents wasted more than 40%. Moreover, 86.9% of respondents answered that they would throw leftover food directly into the trash, only 26 respondents would save leftovers for their next meal, and others chose to feed their food to small animals. Interestingly, almost all respondents had received food-saving information through different channels.

Respondents were categorized by gender, monthly household income level, family source, farm work experience, frequency of online food ordering, and whether they were vegetarians to analyze the distribution of food waste behaviors. Females were found to waste more food than males. Overall, 64.1% of women reported that more than 20% of the food they ordered was wasted, compared to 37.7% of men. Females in our study expressed that a mismatch exists between their smaller appetites and the standardized amount of food they ordered. Ultimately, females felt they lacked choice and price incentives to purchase smaller portions.

**Figure 3:**
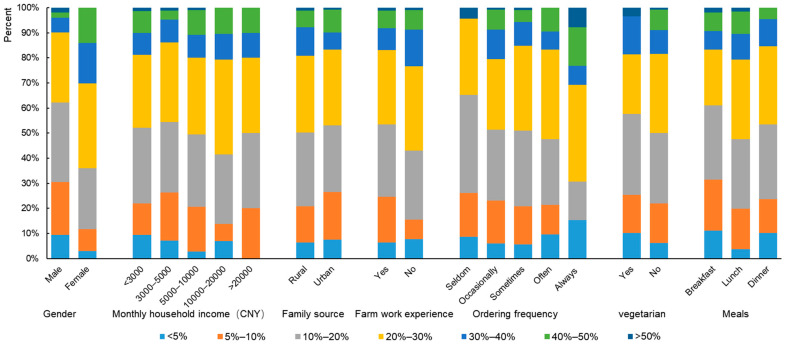
The amount of food waste in different categories of groups.

The amount of food waste respondents estimated seemed less related to household income and family source (urban or rural). However, the group who did not participate in farm work experience (366) reported more waste, with 23% reporting that more than 30% of the food they ordered was wasted. In another group, this percentage was only 16.9%. In addition, food waste tended to be more serious in the group of people who ordered food online more frequently. Finally, the amount of food waste was slightly greater for lunch meals and among non-vegetarians. The details are shown in Figure 3.

### 4.2. Reliability, Validity, and Model Fit

The data were analyzed for reliability using SPSS 25.0. The Cronbach’s alpha coefficient of the model was 0.840, indicating good internal consistency for the total scale. CFA analysis was conducted using AMOS 23.0. After excluding items with low factor loadings, three or more items were retained for each variable. All items in the CFA loaded significantly (*p* < 0.001) on their corresponding factors and had factor loadings above 0.60, which provides evidence for convergent validity. The average variance extracted (AVE) and construct reliability (CR) were higher or equal to the thresholds of 0.50 and 0.70 (Table 2), while over-consumption behavior had an AVE slightly lower than 0.50 but higher than 0.36, which is acceptable. Our results provide support for convergent validity; the details are shown in Table 2. Finally, the discriminant validity was tested and is shown in Table 3. Overall, we found acceptable discriminant validity between the constructs in our study. Finally, goodness of fit indices were tested in AMOS. Important indices of our model fit met the fitness criteria (CMIN/DF = 2.995, RMSEA = 0.064, GFI = 0.902, AGFI = 0.874, PGFI = 0.702, NFI = 0.914, IFI = 0.941, TLI = 0.931, CFI = 0.941).

### 4.3. Hypothesis Testing

**Figure 4:**
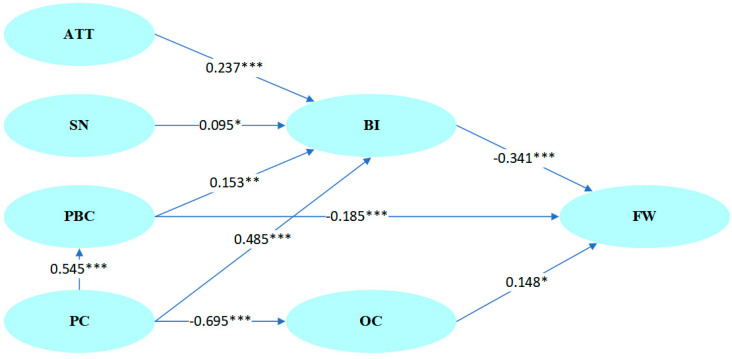
Results of the hypothesized relationships in the SEM model. (* *p* < 0.05; ** *p* < 0.01; *** *p* < 0.001.)

Relationships between variables were tested via SEM. The maximum likelihood estimation results indicated an adequate data fit (Comparative Fit Index, CFI = 0.941, Root Mean Square Error of Approximation, RMSEA = 0.064). The results of the hypothesis testing are shown in Figure 4. These results indicate that attitude, subjective norm, perceived behavioral control, and price consciousness are positively related to behavioral intention to reduce food waste, with coefficients of 0.237 (*p* < 0.001), 0.095 (*p* < 0.05), 0.153 (*p* < 0.01), and 0.485 (*p* < 0.001), respectively. Additionally, price consciousness was positively related to perceived behavioral control, with a coefficient of 0.545 (*p* < 0.001). Perceived behavioral control and behavioral intention to reduce food waste were negatively related to food waste behavior, with path coefficients of −0.185 (*p* < 0.001) and −0.341 (*p* < 0.001), respectively. Price consciousness was negatively related to over-consumption behavior, with a coefficient of −0.695 (*p* < 0.001). Finally, over-consumption behavior was positively related to food waste behavior, with a path coefficient of 0.148 (*p* < 0.05). In summary, H1-H9 were supported.

### 4.4. Mediation Analysis

To examine mediating effects, this study used bootstrapping under a 95% confidence interval with 5000 bootstrap samples, following Taylor et al. [81]. Additionally, we calculated the confidence intervals of the lower and upper bounds to test the indirect effects. Firstly, we ran the test by excluding PBC, BI, and OC from the model to ensure that the direct effects of ATT, SN, and PC on FW were significant.

The results indicated that SN was not significantly related to FW (Table 4), so we performed a mediation analysis excluding SN. The *p*-values for both ATT (*p* < 0.01) and PC (*p* < 0.001) indicated a significant direct relationship with FW. A bootstrap test was used in our comprehensive model to examine whether the indirect effects of BI, OC, and PBC on FW are significant. According to the bootstrap test results (Table 5), both BI and PBC have significant indirect effects on FW (*p* < 0.01), while OC has no significant indirect effect (*p* > 0.05). We used VAF to determine the mediation effect (VAF = indirect effect/total effect; if VAF > 80%, there is a full mediation; if 20% ≤ VAF ≤ 80%, there is partial mediation; and if VAF < 20%, there is no mediation) [82]. Table 6 showed the indirect and total effects of ATT, PBC, and PC on FW.

## 5. Discussion

The results of this study help us better understand the determinants of the food waste behavior related to online food ordering among young adults in China. The model performed as expected and was very explanatory for real problems. A significant positive relationship was found between attitude and behavioral intention to reduce food waste, which is consistent with some previous research findings [25,78]. Moreover, BI showed full mediation between ATT and FW. Attitudes towards reducing food waste involve economic attitudes, environmental attitudes, and moral attitudes [65]. If consumers regard food waste behavior as irrational and less environmentally friendly, these perceptions can evoke their emotions and facilitate the intention to avoid waste; such attitudes can eventually change human behavior. Some consumers who have a very positive attitude towards the intention of reducing food waste will also prevent others from wasting food.

Although subjective norms were found to significantly promote behavioral intention to reduce food waste, they did not show a significant effect on wasteful behavior, suggesting that a behavioral intention to reduce food waste is not a mediator of subjective norms or wasteful behavior. The impact of subjective norms on food waste behavior has been controversial, as some studies suggest an effect [22,66], while others argue the opposite [63,69]. The concepts of group orientation and saving face are also applicable in explaining the behavior of Chinese consumers who revere Confucianism. Saving face can increase the probability of wasting food by reducing the reuse of leftovers, and group conformity can have a significant effect on the ordering of small portion sizes [25]. Chinese dining culture is a key factor informing food waste behavior [24]. On the other hand, the family traditions and social atmosphere of “being thrifty and saving food” will implicitly reduce consumers’ willingness to waste and wasteful behaviors. In our study, consumers who were younger and lived away from home were shown to have weaker perceptions of food conservation, as such consumers are less subject to intervention by their family and elders. In addition, when such consumers choose to order food, they often eat with friends or roommates. Therefore, self-respect in front of their peers may also make younger consumers more wasteful. For example, although our campus engages in various campaigns to save food and reduce waste, which can enhance consumers’ subjective norms, improving other factors, such as perceived behavioral control and price awareness, may be more important to convert food waste reduction intentions into action.

Perceived behavioral control plays an important role in our model. In addition to its significant effects on food waste behavioral intention and behavior, it also has a partial mediating effect between price consciousness and food waste behavior. Our study measured the difficulty of reasonable ordering, individual self-control, and difficulty of leftover disposal. The mean value of the latent variable was low, and many respondents reported that the quantity and quality of the food purchased when ordering online did not match their needs. Moreover, there is large information asymmetry when ordering food, such as the provision of too many staples, cold meals, and poor taste. A lack of storage space and the need to heat leftovers might make such consumers dispose of their food, while stronger self-control can make consumers finish food that does not match their desired tastes [69]. Raising price consciousness can also help improve self-control in reducing waste, but self-control alone is not enough. Consumers also need external support, good ordering services, and relevant information about the food they ordered to enhance perceived behavioral control.

In our research, over-consumption behavior was found to be negatively related to price consciousness and positively related to food waste behavior but was not found to be a mediator of price consciousness or wasteful behavior. Financial loss due to over-consumption of food and food waste is a key issue [83,84] that also provides ideas for interventions to reduce food waste. Moreover, hospitality [34] and self-respect [18] cause consumers to purchase more food when at home or dining out. In the present study, the reasons for consumers’ over-consumption behaviors in online food ordering differed from those in previous studies and were found to stem more from large standard portion sizes, minimum delivery amounts, merchant discounts, and curiosity at the time of purchase, all of which can promote food waste behavior. Additionally, people with stronger price consciousness tend to search for information about food when ordering and plan rationally by gathering information on taste, portion size, and price. These individuals are not prone to over-consumption. In our case, the pathway through which price consciousness influenced food waste behavior was primarily BI or PBC, not OC. Therefore, although consumers with stronger price consciousness are less likely to order more food online, whether or not the food they order is wasted depends largely on PBC and BI.

Price consciousness significantly promotes one’s perceived behavioral control and behavioral intention to reduce food waste. Food waste has positive income elasticity [18,32], and consumers’ consumption decisions on whether, or how much, food to waste are to some extent related to price consciousness. Food waste can lead to property loss. How consumers assess and dispose of such loss is related to current income and expenditures on the one hand and price consciousness on the other [85]. The primary motivation for people with stronger price consciousness to reduce waste is to save money [86], so such consumers reduce food waste by having a stronger sense of PBC and BI. Thus, our findings support the argument that not only consumers’ psychological factors but also retailers’ actions are important factors in reducing food waste [87]. Moreover, some business strategies and environmental factors that are important constraints to food waste avoidance affect consumers’ everyday life decisions [70,80].

Overall, OC was found to be positively related to FW. Additionally, ATT, PBC, and PC were determined to be important factors when considering the paths through which the mediation of BI influences FW. However, price consciousness influenced food waste behaviors primarily through BI or PBC, not OC. Models of consumers’ food waste behaviors, therefore, should consider attitudes, perceived behavioral control, and consumers’ price consciousness. In addition, subjective norms that promote consumers to reduce waste (e.g., food education and cherish food campaigns) and social pressures that drive consumers to waste food (e.g., face-culture) should be considered to identify their impact logistics.

## 6. Conclusions

Food waste is becoming a potential threat to food security, creating a need to enhance awareness of saving food for all and advocating for a simple, moderate, green, and low-carbon lifestyle. Since ordering food from restaurants through the use of delivery apps is a fast-growing area in the hospitality industry in China, reducing food waste in this sector could make a significant difference. From the perspective of consumers, our research partially answers the question of why waste is generated in online food ordering and how that waste can be reduced.

The results of descriptive statistics showed that the food waste situation of young consumers’ online food ordering is very serious. About half of the respondents reported wasting more than 20% of their food, and 18.5% wasted more than 30%. Consumers without farming experience and females wasted more when online ordering food. Some of China’s young people grew up in rural areas and thus had the experience of following their parents to participate in farming work from a young age. Even when they become adults, such individuals may return to their hometowns to help their parents in farming during busy periods. The experience of farming or difficult financial situations during childhood may lead these young consumers to form stronger habits of valuing food than urban-raised consumers. In addition, females in this study reported more waste through eating less, losing weight, etc. Additionally, consumers who order more frequently wasted more food on average.

Our study also found that the level of consumers’ perceived behavioral control was lower than other factors, indicating that it is more difficult for such consumers to reduce food waste under the impacts of various factors such as time, money, dining environment, and obtained information. For consumers ordering food online, perceptual behavioral control is somewhat inhibited by standardized portions and undesirable conditions for storing and heating leftovers.

Based on our research, we believe that the extended TPB model incorporating over-consumption behavior and price consciousness can verify the decision factors for food waste behaviors among young Chinese consumers. The results showed that the various factors promoting consumers’ food waste behaviors when engaging in online food ordering are intertwined. For example, although factors such as consumers’ attitude, subjective norms, perceived behavioral control, and price consciousness can significantly contribute to behavioral intention to reduce food waste, the strength of the effect to reduce food waste behavior may be related to external factors such as catering service approach, food disposal convenience, and marketing strategies. The psychological variables for consumers were closely related to the external conditions and environment. ATT, PBC, and PC are important factors that mediate the influence of BI on FW. For some reason, SN does not significantly influence ultimate waste behaviors through BI. Therefore, a boost to SN through an information campaign or significant personal impacts may improve BI but, at the same time, not further reduce waste. Substantial reductions in food waste can only be achieved through changes in other factors such as increasing the TPB for waste reduction. In our study, price consciousness influenced food waste behavior primarily through BI or PBC, not OC. The reason for this result may be that PC can also lead to OC and greater FW through a propensity to accept “special offers” and similar discounts, which is a research avenue worthy of a follow-up study. In future research, more influencing factors should be considered to identify the determinants of food waste behaviors in online food ordering, such as face culture, dietary knowledge, online marketing strategies, etc.

## 7. Policy Implications

The habits that college students developed on campus may continue to influence their food waste behaviors once they enter the workforce. With the development and prevalence of online-to-offline (O2O) food delivery service, more and more people order food takeaways via application procedures (APPs) and have their meals in workplaces. Furthermore, with the impact of COVID-19, reducing consumers’ food waste in online food ordering has been regarded as an important factor for achieving sustainable food consumption in catering industry. For online food ordering, the take-out food supply system is more complex than other food consumption situation, such as more serious information asymmetry problems in the consumption process, higher costs of reusing surplus food due to lack of infrastructure, and difficulty in achieving on-demand allocation of standardized food, making the take-out food supply system an important factor driving food waste. Employing effective policy measures to optimize the take-out food supply system and convince people to reduce food waste will be key to future sustainable food consumption [88].

The level of food waste in online food ordering can be reduced by optimizing the catering take-out food supply system and promoting lifestyle transformation among residents. From the perspective of policies and regulations, normative service standards should be formulated and promulgated, and smaller meals should be promoted for the online catering industry. A long-term mechanism for storing, heating, and reusing leftover food in different places(such as campus, working places) should also be developed. Evaluation and feedback systems for assessing the quality, taste, hygiene, nutrition, and other information related to the restaurant’s meals should be improved to enhance perceived behavioral control among consumers. In addition, education on sustainable food consumption and shopping planning strategies and knowledge about the resource and environmental impacts of food production should be strengthened to provide consumers a stronger perception of reducing food waste [5] and its related issues such as carbon emissions and the inefficient use of land, water, and labor [39,89,90]. This information will help promote moral responsibility among consumers, and thus change their attitudes and behaviors to reduce waste.

The information asymmetry between consumers and “platforms and merchants” leads to over-consumption and food waste. In addition, the lack of food reuse infrastructure in the take-out food consumption space makes it difficult for consumers who are willing to avoid waste. The management of food waste in take-out restaurants cannot be limited to educating residents, but also to suggesting optimization strategies based on the interaction between online food ordering behavior and the food supply system. Consumers should be encouraged to participate in agricultural practices to raise awareness and responsibility for the food production and the environment. For restaurant industry merchants and platforms, improving online food ordering services and meal delivery modes (such as small portions) could enhance consumers’ perceived behavioral control and thereby reduce waste by presenting appropriate information interventions on the outer packaging. This information would remind consumers to “reduce waste and save resources” and could be supplemented by offering smaller meals and decreasing minimum delivery amounts. In online marketing, we could remind consumers of the quantity, taste, and nutritional content of the meals and establish appropriate marketing activities (e.g., improved discounts or voucher strategies) to reduce food waste in online food ordering.

## 8. Limitations and Future Research

Although our research has some interesting findings, but there are some limitations. Firstly, the food waste behavior of online food ordering in our study was relayed on self-report which may be deviate from reality. The measurement of food waste has been a challenge in the current research on this topic [91] and the main quantitative methods based on first-hand data are diary, direct measurement, survey(by questionnaire), waste composition analysis and mass flow analysis [92]. Our study used the survey(by questionnaire) which is currently widely used [93] and the main purpose of which is to collect information about people’s perceptions and behavior of food waste [31,37,94,95]. Other methods, such as direct measurement(weighing) [18,39,96] and material flow analysis [97] seem to be more accurate, and future studies should also test consumers’ food waste behavior through these methods or some experimental scenarios. Big data feedback based on Internet restaurant platforms can also be used for follow-up studies. Secondly, no component analysis was performed in our study. The caloric, energy and environmental impacts are various for different wasted food. It is very essential to analyze the components of wasted food by future researchers.

Finally, the young consumer group is also a limitation in this study. The university student group may have different food consumption and food waste behavior from other groups in online food ordering situation. For instance, college students are more concerned about body image and dieting, which can promote wasteful behavior. Most of their living expenses are paid by their parents rather than their own money, which can make them less price conscious and more prone to impulsive shopping or excessive purchases. Also, students rarely shop for food to cook for the family, which can reduce their perceptions of the sense of valuing food. Therefore, although individuals 20–35 years old are becoming the main consumers of online food ordering, and this group is representative of food waste research in online food ordering, future studies should use other samples to explore the drivers of food waste behavior. We hope this study explains the factors influencing food waste behavior to some extent and led to further discussion on food waste prevention in the catering industry.

## Figures and Tables

**Table 1:** Demographic information and background factors.

Variable	Categories	Frequency	Percent (%)
Gender	Male	276	57.3
	Female	206	42.7
Grade	Freshman	74	15.4
	Sophomore	129	26.8
	Junior	110	22.8
	Senior	81	16.8
	Master’s students	76	15.8
	Doctoral Students	12	2.5
Family Sources	Urban	132	27.4
	Rural	350	72.6
Vegetarian	Yes	59	12.2
	No	423	87.8
Monthly household income (RMB)	Less than 3000	159	33.0
	3000–5000	167	34.6
	5000–10,000	112	23.2
	10,000–20,000	33	6.8
	More than 20,000	11	2.3
Online food ordering Frequency	Seldom	23	4.8
	Occasionally	298	61.8
	Sometimes	106	22.0
	Often	42	8.7
	Always	13	2.7
Last online food ordering cost (RMB)	10–15	125	25.9
	15–20	149	30.9
	20–30	203	42.1
	More than 30	5	1.0
Last online food ordering food waste (FW1)	Less than 5%	32	6.6
	5–10%	76	15.8
	10–20%	138	28.6
	20–30%	147	30.5
	30–40%	49	10.2
	40–50%	35	7.3
	More than 50%	5	1.0
Total		482	

**Table 2:** Results of Confirmatory Factor Analysis (N = 482).

Factors and Items	Variable Names	Factor Loadings	CR	AVE
Attitude (ATT) [45,63,69]			0.897	0.745
It will make a great contribution to environmental protection for everyone to reduce food waste	ATT1	0.930		
There are still many people in the world who are hungry, and it is immoral if we waste food	ATT2	0.862		
Reducing food waste is a wise choice	ATT3	0.792		
*Scale: strongly disagree(1) to strongly agree (7)*				
Subjective norm (SN) [45,78]			0.891	0.734
People who are important to me do not approve of my excessive ordering	SN1	0.845		
Students and friends around me always eat up the food on their plates to reduce food waste	SN2	0.972		
Most people in my family will pay attention to cherishing food	SN3	0.736		
*Scale: strongly disagree (1) to strongly agree (7)*				
Perceived behavioral control (PBC) [78]			0.911	0.774
It’s not difficult for me to order the right amount of food as I need	PBC1	0.875		
Even if I don’t like the food I get, I try to eat it	PBC2	0.934		
I always can share or reuse the leftovers	PBC3	0.827		
*Scale: strongly disagree (1) to strongly agree (7)*				
Price consciousness (PC) [43,64,79]			0.895	0.741
If I order food and it goes to waste, it’s more than I can afford	PC 1	0.889		
Waste food means waste money	PC 2	0.916		
I consider the price when choosing a meal to make the most cost-effective choice	PC 3	0.770		
*Scale: strongly disagree (1) to strongly agree (7)*				
Over-consumption behavior (OC) [34,45]			0.725	0.468
I always order more food than I need (I don’t plan my purchases when buying food online or offline)	OC 1	0.682		
I always order more food because it is difficult to judge whether the taste meets my needs	OC 2	0.709		
I will order more food online because of discounts (sales, starting delivery amount)	OC 3	0.661		
*Scale: strongly disagree (1) to strongly agree (7)*				
Behavior intention to reduce food waste (BI) [66]			0.831	0.551
I intend to value food and order meals wisely	BI1	0.685		
I intend to use all the leftovers	BI2	0.754		
I want to eat up the meals I order	BI3	0.787		
I intend to notify my friends, family and neighbors to reduce their food waste	BI4	0.740		
*Scale: strongly disagree (1) to strongly agree (7)*				
Food waste behavior (FW) [22,25,80]			0.853	0.663
How much of the food was thrown away when you ordered online last time?	FW1	0.891		
On average, how much of the food ordered online is not eaten up?	FW2	0.872		
How many edible staples are thrown away in meals ordered from apps such as Meituan and Eleme for you?	FW3	0.660		
*Scale: 1. less than 5%; 2.5–10%; 3. 10–20%; 4.20–30%; 5.30–40%; 6. 40–50%; 7. More than 50%*				
**Goodness of fit indeces:** CMIN/DF = 2.995, RMSEA = 0.064, GFI = 0.902, AGFI = 0.874, PGFI = 0.702, NFI = 0.914, IFI = 0.941, TLI = 0.931, CFI = 0.941 **CR** = construct reliability; **AVE**= average variance extracted.				

**Table 3:** Discriminant validity for the measurement model.

	AVE	ATT	PC	SN	PBC	BI	OC	FW
ATT	0.745	0.863						
PC	0.741	0.468	0.861					
SN	0.734	0.376	0.458	0.857				
PBC	0.774	0.379	0.540	0.223	0.880			
BI	0.551	0.558	0.718	0.438	0.523	0.742		
OC	0.468	−0.323	−0.691	−0.317	−0.373	−0.497	0.684	
FW	0.663	−0.309	−0.448	−0.238	−0.420	−0.512	0.387	0.814

**Table 4:** Structure model results excluding PBC, BI and OC.

	Estimate	*p* Values
ATT→FW	−0.188 **	0.007
SN→FW	0.011	0.869
PC→FW	−0.304 ***	0.000

** *p* < 0.01; *** *p* < 0.001.

**Table 5:** Results of bootstrap test.

	Point Estimate	Product of Coefficients		Bootstrapping					
				Bias-Corrected Percentile 95% CI			Percentile 95% CI		
		S.E.	Z	Lower	Upper	Two-Tailed Significance	Lower	Upper	Two-Tailed Significance
ATT→BI→FW	−0.104 ***	0.039	−2.667	−0.180	−0.051	0.000	−0.173	−0.047	0.001
PBC→BI→FW	−0.045 **	0.020	−2.250	−0.086	−0.018	0.003	−0.082	−0.015	0.005
PC→BI→FW	−0.208 ***	0.065	−3.200	−0.332	−0.115	0.000	−0.325	−0.110	0.001
PC→OC→FW	−0.130	0.082	−1.585	−0.272	−0.003	0.093	−0.265	0.003	0.108
PC→PBC→FW	−0.127 **	0.044	−2.886	−0.203	−0.059	0.002	−0.200	−0.056	0.002
PC→PBC→BI→FW	−0.036 **	0.016	−2.250	−0.068	−0.014	0.003	−0.065	−0.012	0.005

** *p* < 0.01; *** *p* < 0.001.

**Table 6:** Indirect and total effects of ATT, PBC and PC.

Path	Point Estimate		*p* Values	Results
ATT→BI→FW	Indirect effect	−0.104 ***	0.000	Full mediation
ATT→FW	Total effect	−0.104 ***	0.000	
PBC→BI→FW	Indirect effect	−0.045 **	0.003	Partial Mediation
PBC→FW	Total effect	−0.204 ***	0.000	
PC→BI→FW	Indirect effect	−0.208 ***	0.000	Partial Mediation
PC→OC→FW	Indirect effect	−0.130	0.093	No Mediation
PC→PBC→FW	Indirect effect	−0.127 **	0.002	Partial Mediation
PC→PBC→BI→FW	Indirect effect	−0.036 **	0.003	No Mediation
PC→FW	Total effect	−0.501 ***	0.000	

** *p* < 0.01; *** *p* < 0.001.

## Data Availability

Data was available contained within the article.
